# RETRACTION: The Outcome of Sutured Wounds Compared With Tissue Adhesive for Paediatric Wound Closure: A Meta‐Analysis

**DOI:** 10.1111/iwj.70210

**Published:** 2025-02-02

## Abstract

**RETRACTION:** CuiX. , ZhangY. , WangN. , ChenY. , XuJ. , and HouJ. , “The Outcome of Sutured Wounds Compared with Tissue Adhesive for Paediatric Wound Closure: A Meta‐Analysis,” International Wound Journal 20, no. 8 (2023): 3298–3306, 10.1111/iwj.14210.37221969 PMC10502276

The above article, published online on 23 May 2023, in Wiley Online Library (http://onlinelibrary.wiley.com/), has been retracted by agreement between the journal Editor in Chief, Professor Keith Harding; and John Wiley & Sons Ltd. Following an investigation by the publisher, all parties have concluded that this article was accepted solely on the basis of a compromised peer review process. The editors have therefore decided to retract the article. The authors did not respond to our notice regarding the retraction.

**RETRACTION:** X. Cui , Y. Zhang , N. Wang , Y. Chen , J. Xu , and J. Hou , “The Outcome of Sutured Wounds Compared with Tissue Adhesive for Paediatric Wound Closure: A Meta‐Analysis,” International Wound Journal 20, no. 8 (2023): 3298–3306, 10.1111/iwj.14210.37221969 PMC10502276

The above article, published online on 23 May 2023, in Wiley Online Library (http://onlinelibrary.wiley.com/), has been retracted by agreement between the journal Editor in Chief, Professor Keith Harding; and John Wiley & Sons Ltd. Following an investigation by the publisher, all parties have concluded that this article was accepted solely on the basis of a compromised peer review process. The editors have therefore decided to retract the article. The authors did not respond to our notice regarding the retraction.

